# Patients’ and clinicians’ perspectives on the primary care consultations for acute respiratory infections during the first wave of the COVID-19 pandemic: an eight-country qualitative study in Europe

**DOI:** 10.3399/BJGPO.2021.0172

**Published:** 2022-04-20

**Authors:** Marta Wanat, Melanie Eugenie Hoste, Nina Helene Gobat, Marilena Anastasaki, Femke Böhmer, Slawomir Chlabicz, Annelies Colliers, Karen Farrell, Sophie Hollerbach, Maria-Nefeli Karkana, John Kinsman, Christos Lionis, Ludmila Marcinowicz, Katrin Reinhardt, Ingmarie Skoglund, Pär-Daniel Sundvall, Akke Vellinga, Herman Goossens, Christopher C Butler, Alike van der Velden, Sibyl Anthierens, Sarah Tonkin-Crine

**Affiliations:** 1 Nuffield Department of Primary Care Health Sciences, University of Oxford, Oxford, UK; 2 Department of Family Medicine and Population Health, University of Antwerp, Antwerp, Belgium; 3 Laboratory of Medical Microbiology, Vaccine & Infectious Disease Institute, University of Antwerp, Antwerp, Belgium; 4 Clinic of Social and Family Medicine, Faculty of Medicine, University of Crete, Crete, Greece; 5 Institute of General Practice, Rostock University Medical Centre, Rostock, Germany; 6 Department of Family Medicine, Medical University of Bialystok, Bialystok, Poland; 7 School of Medicine, National University of Ireland, Galway, Ireland; 8 European Centre for Disease Prevention and Control, Solna, Sweden; 9 Department of Obstetrics, Gynaecology and Maternity Care, Medical University of Bialystok, Bialystok, Poland; 10 General Practice/Family Medicine, School of Public Health and Community Medicine, Institute of Medicine, Sahlgrenska Academy, University of Gothenburg, Sweden, Sweden; 11 Research, Education, Development & Innovation, Primary Health Care, Region Västra Götaland, Sweden; 12 HRB Primary Care Clinical Trials Network, Ireland; 13 Laboratory of Clinical Microbiology, Antwerp University Hospital, Edegem, Belgium; 14 National Institute for Health Research Health Protection Research Unit in Healthcare Associated Infections and Antimicrobial Resistance, University of Oxford in Partnership with Public Health England, Oxford, UK; 15 Julius Center for Health Sciences and Primary Care, University Medical Center Utrecht, Utrecht, the Netherlands

**Keywords:** primary health care, remote consultations, telemedicine, COVID-19

## Abstract

**Background:**

The impact of the COVID-19 pandemic on patients’ and clinicians’ perceptions of healthcare-seeking behaviour and delivery of care is unclear. The pandemic accelerated the use of remote care, and understanding its benefits and drawbacks may inform its implementation during current and future healthcare emergencies.

**Aim:**

To explore patients’ and primary care professionals’ (PCPs) experiences of primary care delivery in the first wave of the pandemic.

**Design & setting:**

Qualitative study using semi-structured interviews in primary care in eight European countries (England, Ireland, Belgium, the Netherlands, Greece, Poland, Sweden, and Germany).

**Method:**

A total of 146 interviews were conducted with 80 PCPs and 66 patients consulting for respiratory tract infection (RTI) symptoms, in eight European countries. Data were collected between April and July 2020, and analysed using thematic analysis.

**Results:**

It was found that patients accepted telemedicine when PCPs spent time to understand and address their concerns, but a minority preferred in-person consultations. PCPs felt that remote consultations created emotional distance between themselves and patients, and they reported having to manage diverse COVID-19-related medical and social concerns.

**Conclusion:**

Remote consultations for RTI symptoms may be acceptable long term if both groups are happy to use this format, but it is important that PCPs take time to address patients’ concerns and provide safety-netting advice.

## How this fits in

When considering the benefits and drawbacks of remote consultations, it is important to consider patients’ underlying concern leading to seeking help, among other factors; yet there is limited understanding of patients’ and PCPs’ experiences of consulting and providing care for respiratory symptoms during the COVID-19 pandemic. This gap has been addressed by conducting interviews with patients and PCPs in eight European countries. It was found that patients’ level of confidence in their clinician remained unchanged during remote consultation, if both patients and PCPs were happy to use this format, PCPs took time to address patients’ concerns, and the importance of the doctor–patient relationship was considered. In addition, PCPs across all countries described having to deal with diverse COVID-19-related queries that went beyond medical advice and were difficult to manage owing to limited time and guidance. This study highlights the need for a flexible approach in the delivery of remote consultations as well as resources for primary care to direct people to access other support where appropriate.

## Introduction

Primary care rapidly adjusted delivery of care at the onset of the COVID-19 pandemic. This often meant operating a ‘closed-door‘ policy and switching to remote consultations.^1–3^ In most European countries, remote consultations were either not or seldom offered pre-pandemic,^4^ but the pandemic necessitated adoption of new approaches, changing the management of patients.

Pre-pandemic studies on patients and PCPs highlighted both benefits and drawbacks of remote consultations.^5,6^ The COVID-19 pandemic provides a new context where identified benefits and drawbacks of these consultations need to be re-examined^7^ for patients and PCPs. A growing number of studies explored patients’ experiences of receiving remote care during the pandemic, including patients with dementia,^8^ heart failure,^9^ or mental health problems,^10^ and migrants,^11^ highlighting both challenges and opportunities. While some of these challenges may be seen across a number of conditions, reason for consultation is an important factor to be considered when deciding whether they may be beneficial and appropriate.^12,13^

Studies conducted with clinicians during the COVID-19 pandemic found that they tried to provide patient-centred care during remote consultations by taking time to listen to patients’ concerns and providing relevant and appropriate reassurance.^14^ However, with time, PCPs found them to be more time-consuming, clinically challenging, and less satisfying.^3^ This has been further magnified by the uncertainty in assessing respiratory and/or related COVID-19 symptoms remotely.^15,16^ No studies explored patients’ and PCPs’ experiences of consulting and providing care for respiratory symptoms during the pandemic, illustrating a need to understand their experiences when dealing with symptoms that could be caused by COVID-19. This gap is addressed by exploring patients’ and PCPs’ views of consultations for these symptoms, focusing on healthcare-seeking behaviour and consultation processes, during the first wave of the COVID-19 pandemic.

## Method

### Setting

This was a study conducted in primary care. Key differences between primary care settings have been described elsewhere.^17^ To summarise, during the time of the data collection, in all countries, patients accessing primary care were triaged before they were able to come into the surgery.^17^ In most countries, triage was carried out over the telephone by either a PCP or a receptionist, except in Greece and Sweden where triage took place by the practice door.^17^ The majority of patients with RTI symptoms had their consultations with a PCP over the telephone.^17^ Countries such as England, Belgium, Ireland, and the Netherlands later established COVID-19 hubs where patients with RTI symptoms could be seen by a PCP.^17^ Before the pandemic, telephone consultations were possible in all countries except in Belgium.^17^

Availability of testing was limited during time of interviews, with the majority of countries in Europe struggling to keep up with the demand in testing in the first wave of the pandemic.^18^ All countries included in this study faced restricted testing capacity and, as a result, testing was limited to patients with severe RTI symptoms and/or for patients who were hospitalised.^18–26^

### Study design and participant recruitment

A qualitative study was conducted and patients and PCPs were recruited from eight European countries: England, Belgium, the Netherlands, Ireland, Sweden, Poland, Greece, and Germany during the first wave of the COVID-19 pandemic.^27^ Participants were recruited through an existing clinical European primary care network, where a network coordinator from each country had access to a number of primary care sites.^28^

Using both convenience and purposeful sampling, it was aimed to recruit a range of patients and PCPs. The inclusion criteria for PCPs (for example, doctors and nurses) were: 1) delivering care for patients presenting with RTI symptoms in primary care services; and 2) having worked in primary care for >12 months. Inclusion criteria for patients include having consulted face-to-face or remotely, with RTI symptoms (regardless of whether they overlapped with COVID-19 symptoms) in primary care. As the aim was to understand how patients with RTI symptoms may seek help; these symptoms were defined as typical symptoms of RTI infections, rather than COVID-19. Following a consultation, patients were identified by network coordinators (GPs) not involved in their consultation, and were approached for an interview via email, text, or face-to-face within 2 weeks of their consultation. If they were interested in taking part, they were asked to contact the local researchers, who were not part of the clinical team. Parents were invited when patients were aged <16 years. It was aimed to recruit 8–15 participants per country in each group. Additional information on patients’ and clinicians’ characteristics by country can be found in Supplementary Tables S1 and S2, respectively.

### Interviews

Nine experienced qualitative researchers from the existing clinical European primary care network completed the interviews. Two semi-structured interview guides were developed based on the study aims: one for patients and one for PCPs (see Supplementary Boxes S1 and S2). Interviews took place by telephone or in-person. All participants provided oral or written informed consent. Interviews were audiorecorded, transcribed verbatim, and translated into English where necessary.

### Data analysis

Data collection and analysis took place concurrently. Patient and PCP interviews were analysed separately by two authors using a combination of deductive and inductive thematic analysis.^29^ Transcripts from England, Belgium, the Netherlands, and Sweden were read line-by-line and were coded deductively into a priori frameworks based on the topic guides and agreed by the core research team. Data within each framework category was coded inductively to create subcategories. They were then grouped to form subthemes and themes. The remaining transcripts from other countries were read line-by-line and coded into these thematic frameworks. This process involved an iterative and consensus-based approach, drawing on a constant comparison method^30^ to identify similarities and differences across interviews and countries. Data triangulation was used by comparing patients’ and PCPs’ datasets to further understand key similarities and differences in relation to the study aim.^31^ At each stage of this process, data were discussed within the study team to ensure rigour, while the ongoing analysis was discussed within the multidisciplinary study team and with all interviewers. This was done for each country on a monthly basis to understand local contexts and, where relevant, to interpret findings. NVivo (version 12) was used to facilitate data analysis.

## Results

A total of 146 interviews were conducted; 66 with patients and 80 with PCPs. Patient interviews were conducted between 6 April and 29 July 2020, and PCP interviews were conducted between 2 April and 2 July 2020. Interviews in England, Belgium, the Netherlands, and Ireland took place during full lockdown, while interviews in other countries took place between 1 week and 10 weeks after restrictions eased. Patient interviews lasted between 14 minutes and 55 minutes (mean 29 minutes) and PCP interviews between 17 minutes and 86 minutes (mean 35 minutes).

A total of 11 themes were identified. The following seven themes were related to patients: 1) patients’ experiences of being unwell; 2) significance of SARS-CoV-2 testing; 3) views on consultations with healthcare services; 4) impact of the pandemic on daily life; 5) strategies for prevention of SARS-CoV-2 transmission; 6) perceptions of media reporting of COVID-19; and 7) views on participating in scientific research. The following four themes were related to PCPs: 1) PCPs‘ sense of personal risk;^32^ 2) PCPs’ views of COVID-19 testing;^32^ 3) transformation of primary care delivery and PCPs‘ experiences of these changes;^17^ and 4) navigating a new relationship with patients. This article reports data on two themes and their related subthemes, one each for patients and PCPs. These themes, ‘views on consultations with healthcare services‘ from the patients’ dataset and ‘navigating a new relationship with patients‘ from the PCPs’ dataset, focus on key issues related to how patients and PCPs have experienced receiving and delivering care in primary care, and have been managing RTI symptoms during the pandemic. Together they provide insight into barriers and facilitators to implementing remote care in relation to RTI. Remaining themes are described elsewhere.^17,32^ Quotes are presented within results and additional supporting quotes are presented in Supplementary Tables S3 and S4.

### Patient results: Views on consultations with healthcare services

#### Subtheme 1: Perceived need for consulting a PCP

Across all countries, patients reported different reasons for wanting to consult their PCP, such as concerns about RTI symptoms, wanting to know if they had contracted COVID-19, and/or requesting a sick note. Notably, in all countries, patients with persistent RTI symptoms, and/or patients who were in at-risk groups, were extremely worried if they had COVID-19. This prompted them to contact a PCP to understand the cause behind their symptoms and how to manage them:

*‘I was a bit concerned because my doctor had said to me that I was in the at-risk category … when I woke up I was having shortness of breath and it was at that stage I kind of started to panic so I rang my doctor and the receptionist said to me that she would get my doctor to ring me back. He rang me back within two hours*.*‘* (Participant [P]1, Ireland)

In Belgium, the Netherlands, Poland, Germany, Greece, and Ireland, patients could directly contact their PCP. In England and Sweden, patients with RTI symptoms were encouraged to first contact helplines to ease pressure off primary care. Some patients in England and Ireland were initially hesitant to contact a PCP even though some suspected they had COVID-19 as they expected being told to ‘just‘ stay at home, rest, and take paracetamol, while others did not want to burden the healthcare system (see Supplementary Table S3).

Despite their hesitancy, patients still contacted PCPs for advice, reassurance, or had been directed to a clinician through helplines. Particularly in England, patients were mainly dissatisfied with the advice they received via helplines, received conflicting advice, or felt that the call was too short and, ultimately, preferred to speak to their PCPs:

*‘I felt they* [GP] *were actually a lot better than 111, because the GP had explained that the advice that I was given was incorrect, that because of my asthma, that I could actually ring an ambulance and they were a lot more, as I said, more informative and more* […] *try and keep me out of hospital the best they could, but was explaining the risks and things to look out for and deterioration signs, stuff that I never got from 111, which I found then very supportive*.*‘* (P8, England)

#### Subtheme 2: Level of confidence in PCP remained unchanged by remote consultation

There was a mix of consultation formats across all countries, with the majority conducted remotely via telephone or video, and some in-person. In all countries, facilitators to accepting remote consultation included understanding the reason behind the change in consultation format and having the space to have their concerns addressed. Patients generally understood that in-person consultations were not possible owing to restrictions. They also reported that PCPs took time to understand their symptoms, encouraged them to ask questions, gave recommendations, and insisted that patients reach out if their symptoms persisted or worsened, instilling a feeling of reassurance and consequently, satisfaction with the consultation (see Supplementary Table S3).

Importantly, however, a number of patients in Poland and Greece expressed reservations about remote consultations and their impact on quality of care. They would have preferred an in-person consultation as it would include a physical examination and, thus, provide further reassurance and a more concrete diagnosis. Some felt that a remote consultation was not sufficient for diagnosing RTI symptoms, especially those with more severe respiratory and/or COVID-19 symptoms and who were at higher risk, but still appreciated speaking to their PCP:

*‘I contacted a primary care clinic because I had a raised temperature and breathing problems. I was weak, I had difficulties with walking, and I was suffering from very severe muscle pains* […] *I have very low immunity.* […] *All patients were forced to consult by phone. The doctor devoted considerable time to hear me out, to learn about my fears and symptoms and to answer the questions I had. However, it was not the same as going to the clinic and seeing the doctor personally. If I had been allowed to go to the clinic, she would have been able to examine me.’* (P2, Poland)

With access to testing limited at the time of interviews, patients trusted and felt reassured when their PCP said they did not think they had COVID-19 (see Supplementary Table S3). The confidence that patients had in decisions made by their clinician appeared to be strengthened when there was a pre-existing relationship (see Supplementary Table S3). Despite the change in mode of consultation, regardless of a pre-existing relationship, patients still felt cared for and taken seriously:

*‘I definitely appreciate that GP enormously* […] *In that conversation, she asked at least twice whether I had any questions. She was very attentive and took her time with the consultation and ensured that all my questions were answered, so for me that was certainly reassuring*.*‘* (P2, Belgium)

### PCP results: Navigating a new relationship with patients

#### Subtheme 1: Advising on risks related to COVID-19

PCPs in all countries reported that patients worried about their RTI symptoms and sought their advice. PCPs described that they tried to manage these symptoms over the telephone, but often with extensive history-taking, frequent follow-up, and safety netting.

*‘You give them very concrete tips. For example, you say if the fever gets higher or if you’re really short of breath. Or if indoors you have difficulty climbing stairs, that’s the tips that you give, then you should contact us again*.*‘* (P4, GP, Belgium)

In line with patients’ data, PCPs in all countries reported that patients mainly seemed to accept not seeing their clinician and were grateful to receive remote care. To some extent patients were worried about coming into the practice because of the risk of catching COVID-19. In Sweden, where tents had been set up outside practices for patients with signs of infection, PCPs highlighted that patients were concerned about the increased risk of getting infected with COVID-19 by others because of being in contact with other patients with suspected COVID-19:

*‘They don’t always want to go into this tent and be examined* […] *it’s known as “the corona tent”, so they think they’ll be infected as soon as they go in*.*‘* (P5, nurse, Sweden)

Consequently, PCPs felt their role involved encouraging patients to come into the practice or attend outpatient appointments in secondary care, in case of urgent need. They spent time providing explanations for infection prevention procedures, which seemed to reassure patients.

In contrast, some PCPs in Poland, Greece, and Sweden reported that some patients were unhappy about being offered remote consultations and insisted on coming to the clinic during lockdown. Other PCPs also seemed to link this to patients not considering the pandemic as serious:

*‘Some people could not understand that they should not come to the doctor’s office as they did before the coronavirus. That is, they came whenever they wanted, whether they had an appointment or not. There was frustration, some people thought that the doctor did not want to see them, which was not the case!‘* (P5, GP, Greece)

#### Subtheme 2: Primary care as the first point of contact during the pandemic

PCPs highlighted that patients’ queries went beyond managing COVID-19 symptoms and included wanting information on the following: who should be shielding; risk factors for COVID-19; and what symptoms meant in relation to long-term conditions. PCPs felt that they were asked for advice as they were perceived as trustworthy sources of information:

*‘A lot of it is reassurance,* […] *so if their asthma has worsened trying to work out whether it is just a change in symptoms or whether they might have coronavirus.* […] *I think they would rather speak to me about their query than go on a website about it, because it’s a more personal service*.*‘* (P6, nurse, England)

Some PCPs in Sweden, Poland, Greece, Germany, and England also reported that patients called for help in understanding and ‘endorsing‘ public health advice; for example, taking part in social activities or how to implement safety measures. Dealing with these topics meant consultations took longer:

*‘I sometimes have very big problems with time management during telephone consultations because patients really need this contact, conversation, solace, explanation, and the confirmation of what they hear in the media. They need to know if what the media are saying is true and they want to hear it from a doctor they trust*.*’* (P2, GP, Poland)

Interestingly, PCPs in all countries reported that patients also sought advice on a variety of social issues as well, including concerns about the impact of the pandemic on the following: finances when unable to work; not having access to childcare; or going into work because of the risk of catching COVID-19 (see Supplementary Table S4). Consistent with patient data, PCPs felt that patients turned to them for help in managing their fears of getting COVID-19 at work, by requesting a sick note to avoid contact with colleagues. PCPs’ responses to these queries varied; for example, some believed that anxiety around going into work was a valid reason, while others felt that worries about the pandemic were not a reason to issue a sick note:

*‘A lot of people are afraid to go to work, actually. That’s a little awkward, too. Try to motivate the anxious people to go to work because they often come for a sick note while they’re not sick*.*‘* (P6, GP, Belgium)

*‘There’s been quite a lot of work on sick notes, so quite a few people are too scared to go to work. They’re not ill and not on the shielded list but they’re just too frightened, so I’ve been issuing notes with anxiety as the reason for not going to work*.*’* (P9, GP, England)

In addition, PCPs in Germany highlighted the lack of guidance on whether patients should be issued with sick notes in such situations (see Supplementary Table S4).

#### Subtheme 3: PCPs adjusting to the changing doctor–patient relationship

PCPs in all countries highlighted that the nature of their relationship with patients had changed. They felt that the new ways of delivering care and somewhat limited contact with patients created an emotional distance. For instance, some PCPs in Ireland described that because of the pandemic, they could not attend patients’ funerals, which they found uncomfortable as it was customary before:

*‘It has also changed the way I interact with people* […] *we have had a few of our patients pass away in the last few weeks.* […] *It’s very unusual for us not to go out to the house and sympathise with the family in person*.*‘* (P1, GP, Ireland)

Some PCPs in Greece commented that they saw face-to-face contact as an essential part of the doctor–patient relationship and their role as a GP; an appointment enabled patients to talk about other issues and was part of their routine, which they also valued.

*‘We here, have another type of contact, closer to a family situation.* […] *So, we missed that, and we missed a lot of our patients. Especially in older patients, communication and human contact are a cornerstone of wellbeing. So, we lost that! And we miss it too*.*‘* (P1, GP, Greece)

### Results triangulation

Interviews highlighted similarities between patients’ and PCPs’ experiences regarding remote consultations. First, patients and PCPs accepted remote consultations for RTI symptoms if they believed it was beneficial for their safety. Second, both patients and PCPs stated the importance of safety netting and follow-up. Specifically, patients emphasised the value of PCPs taking time to listen to their concerns, answering their questions, and checking up on them. This was also evident with PCPs who commented that they tried to care for patients with RTI symptoms by extensive history-taking, frequent follow-up, and safety netting. Finally, both groups also described the importance of the doctor–patient relationship in relation to RTI problems as well as non-medical issues. Patients stressed the significance of feeling listened to, which provided reassurance. Interestingly, the interviews with patients did not seem to suggest that rapport was lacking in their interactions but PCPs worried about treating patients with dignity and empathy, and stated they worked hard on maintaining these aspects. In addition, PCPs reported that patients called them with a variety of social issues and help with understanding public health messages. These were not explored in interviews with patients, but they did highlight that a pre-existing relationship with a GP facilitated further trust, when being offered a remote consultation.

## Discussion

### Summary

To the authors’ knowledge, this is the first study to explore patients’ and PCPs’ views of consultations for RTI symptoms in European primary care settings during the pandemic. It was found that patients accepted remote consultation for RTIs when PCPs spent time to reassure them and address their concerns. However, a minority of patients still preferred in-person consultations. PCPs and patients valued doctor–patient relationships but PCPs, to a greater degree than patients, felt that COVID-19 regulations affected the doctor–patient relationship. PCPs also described having to deal with diverse COVID-19-related queries, which went beyond medical advice and were difficult to manage owing to limited time and guidance.

### Strengths and limitations

This study benefits from interviews with both patients and PCPs, which were conducted within similar time windows within each country, allowing the triangulation of patients’ and PCPs’ perspectives. A number of limitations are also acknowledged. First, the interviews took place at different time points during the first wave of the pandemic, which meant that views on the perceived needs and acceptance of the consultations might have been affected. Second, the study focused on managing RTI symptoms in primary care, rather than exclusively on the use of remote consultations. Third, it is important to acknowledge a great variation not only across countries but also within them, which illustrates the variety of consultation styles at that time in the pandemic; it is possible that more interviews in each country would reveal additional factors affecting patients’ and PCPs’ experiences of receiving and delivering care. Fourth, the initial analysis of data concentrated on England, Belgium, the Netherlands, and Sweden giving a framework to apply to date to other countries. Another method might have emphasised different aspects of the data. However, regular discussions with interviewers and teams from all countries focused on understanding key similarities and differences, ensuring that all countries were equally represented. Using field notes that were kept during data collection also facilitated these discussions. Fifth, owing to the rapid data collection and analysis, it was not possible to iterate topic guides in parallel.

### Comparison with existing literature

Telehealth’s benefits (such as improved access to care,^33,34^ reduced need for travel,^33^ or continuity of care^35^) and drawbacks (such as exacerbating health inequalities for people without access to technology^36^ or not being appropriate when discussing sensitive information^5,6^ or ‘bad news’^5,6^) have been reported before the pandemic. However, the COVID-19 pandemic accelerated^37^ and provided a new context where these barriers and facilitators needed to be re-examined. In line with other studies,^9,38–41^ patients considered the risk in attending face-to-face appointments as an important factor when deciding whether to accept a remote consultation. Specifically, the assessment of risk seemed to involve considering the potential risk of being infected with COVID-19 and the risk of not being seen or physically examined in relation to their condition; this seemed to be especially stressed by patients with severe RTIs. Therefore, remote consultations could be seen as a barrier for patients who need to be physically examined, yet can be a facilitator for the provision of care for patients at risk of being infected by the virus.^42^ Studies with patients with dementia and their carers,^8^ patients with mental health problems,^10^ and migrants^11^ during the pandemic, also highlighted that patients preferred in-person consultations,^8^ thus demonstrating similarities between certain vulnerable groups in terms of their needs for face-to-face care. Patients also emphasised the importance of a pre-existing relationship with their PCP that also facilitated their willingness to accept remote care.^12,43^ This is in line with a great body of literature highlighting the positive effects of continuity of care, including both patient-related outcomes such as satisfaction,^44^ lower use of emergency or urgent care,^45^ and improved health promotion,^46^ as well as clinician-based outcomes such as their job satisfaction.^47^ As identified in the present study, PCPs were seen also as a trusted source of information with the varied queries they received from their patients. This is in line with other studies highlighting that healthcare professionals (HCPs) have been a trusted source of information during this pandemic.^25,48^

PCPs in the present study also highlighted that the doctor–patient relationship changed as a result of the pandemic. On one hand, the limited face-to-face contact with patients presented physical or social barriers to usual ways of conducting consultations, but also in relation to building and maintaining relationships with patients, which they found challenging. Others also highlighted the dangers of remote consultations becoming more transactional, rather than based on the doctor–patient relationship. This is in line with previous studies describing PCPs’ experiences of using personal protective equipment (PPE)^3^ and working with vulnerable populations during the COVID-19 pandemic.^11^ On the other hand, these changes also meant additional stresses for PCPs in trying to manage relationships with patients, which is in line with a growing literature highlighting the negative impact of working during the pandemic on HCPs.^49–52^ The present study emphasises the complexity of changes to the doctor–patient relationship as well as how PCPs perceive their role.

PCPs also reported dealing with more social-related queries in addition to medical ones. A study with PCPs in Belgium described spending a considerable amount of time reassuring patients and answering questions during the first wave.^14^ The present study adds to literature by demonstrating the variety and magnitude of queries on finance, public health messages, managing childcare, or worries about going into work, which added to PCPs’ workload.

### Implications for practice

While remote consultations might be necessary during periods of restrictions and appropriate for straightforward or minor queries, some literature has underlined that the majority of queries in general practice are often not simple.^43^ A recent framework highlighted six areas to be considered when implementing remote consultations: the reasons for consulting; factors related to patient; healthcare organisations including staff and technologies; home and family context; wider system; and finally, the clinical relationship with a GP,^12,13^ illustrated by the present study as well.

First, patients with mild RTI symptoms accepted remote consultations but those with severe RTI symptoms seemed to prefer face-to-face consultations, pointing to the significance of the reason for consulting. While the evidence is still emerging, there are also possible unintended consequences of remote consultations for RTI, including an increased risk of non-evidence-based prescriptions for antibiotics remotely.^53^ Second, patients from certain countries were less willing to accept remote care, highlighting that socio-cultural aspects and patients’ attitudes, not only towards illness but also remote consulting, can influence or explain why they seek help through a particular mode of consultation.^13^ Third, it was found that PCPs and patients valued face-to-face contact, even though it was not always necessary. Helplines for RTI-related queries in the context of the COVID-19 pandemic have not been hugely successful in providing reassurance as patients subsequently preferred speaking to their clinician about their symptoms, indicating the value of the clinical relationship with a GP. Equally, the PCPs in the study also highlighted the limitations of remote consultations in building or maintaining relationships with patients, related to the technology itself, as well as their perceptions related to what their role should involve. This point has also been reflected in the top three priorities set by the Royal College of General Practitioners that asserted the importance of ‘relationship-based care‘, involving taking the time to build and maintain relationships, and trust between doctors and patients.^54^ Finally, it is important to highlight the importance of the context in which remote consultations have been implemented (widely); that is, the COVID-19 pandemic. Patients’ and clinician’s views on what is acceptable and needed going forward will need to be carefully monitored. All together, findings from the present study call for a flexible approach in the delivery of remote consultations.

The present study illustrates that during the pandemic, patients had many queries related to COVID-19 and its consequences, including those non-medical in nature. PCPs are well-placed to support people in the community with the trust placed in them by patients, but not all these queries are within the remit of primary care. One way of reducing pressure related to social needs in primary care is through social prescribing, also known as community referral to social services. It is starting to be implemented in a number of countries in Europe^55,56^ to address patients’ needs holistically and could be an option for future health emergencies, with clear guidance and planning on how these services can be made available. In addition, some of these activities, such as issuing a sick note, also required a lot of PCPs’ time. Ensuring that PCPs can delegate some of these tasks to other professionals might be beneficial in easing their workload.

In conclusion, managing RTI infections during the pandemic might pose challenges for both patients and PCPs in primary care. Remote consultations for RTI symptoms may be acceptable in the long term if both patients and clinicians are happy to use this format. PCPs need to take time to address patients’ concerns and provide safety-netting advice when consulting remotely. Some RTI consultations and patients may not be suited for remote consultations owing to the need or perceived need for physical examinations. Patients’ and PCPs’ preferences and the importance of the doctor–patient relationship should also be considered. Primary care is well-situated to support social needs of patients during healthcare emergency, as PCPs are considered a trusted source of information. However, not all concerns can and should be addressed by PCPs, who need resources to direct patients to access other support where appropriate.

## Data availability

The datasets generated and/or analysed during the current study are not publicly available as the programme of research is continuing and further analysis is ongoing but are available from the corresponding author on reasonable request.
